# A Quantitative Study of a Directional Heat Island in Hefei, China Based on Multi-Source Data

**DOI:** 10.3390/s23063041

**Published:** 2023-03-11

**Authors:** Biao Shi, Lili Tu, Lu Jiang, Jiyuan Zhang, Jun Geng

**Affiliations:** 1 College of Resources and Environment, Anhui Agricultural University, Hefei 230036, China; 2 Jiangsu Provincial Key Laboratory of Geographic Information Science and Technology, International Institute for Earth System Science, Nanjing University, Nanjing 210046, China; 3 School of Geographic and Biologic Information, Nanjing University of Posts and Telecommunications, Nanjing 210023, China; 4 School of Civil Engineering, Hefei University of Technology, Hefei 230009, China; 5 The Centre d’ Etudes Spatiales de la Biosphere, University of Toulouse, 31062 Toulouse, France

**Keywords:** thermal infrared remote sensing, urban surface temperature, surface urban heat island, thermal radiation directionality, Hefei City

## Abstract

Surface urban heat islands (SUHIs) are essential for evaluating urban thermal environments. However, current quantitative studies of SUHIs ignore the thermal radiation directionality (TRD), which directly affects study precision; furthermore, they fail to assess the effects of TRD characteristics at different land-use intensities, on the quantitative studies of SUHIs. To bridge this research gap, this study eliminates the interference of atmospheric attenuation and daily temperature variation factors, in quantifying the TRD based on land surface temperature (LST), from MODIS data and station air temperature data for Hefei (China) from 2010–2020. The influence of TRD on SUHI intensity quantification was evaluated by comparing the TRD under different land-use intensities in Hefei. The results show that: (1) daytime and nighttime directionality can reach up to 4.7 K and 2.6 K, and occur in areas with the highest and medium urban land-use intensity, respectively. (2) There are two significant TRD hotspots for daytime urban surfaces, where the sensor zenith angle is approximately the same as the forenoon solar zenith angle, and where the sensor zenith angle is near its nadir in the afternoon. (3) The TRD can contribute up to 2.0 K to the results of assessing the SUHI intensity based on satellite data, which is approximately 31–44% of the total SUHI in Hefei.

## 1. Introduction

Land surface temperature (LST) is one of the key parameters for studying processes in land surface systems at regional and global scales, enabling monitoring of the spatial and temporal variability in the energy balance state at the surface [1,2,3,4]. Satellite thermal infrared remote sensing is an effective means of obtaining LST [5,6,7,8]. With the rapid development of remote sensing technology and inversion algorithms, LST data obtained by thermal infrared remote sensing inversion algorithms are widely used in various fields, such as evapotranspiration and soil moisture estimation [9], urban heat island studies [10,11,12,13], disaster monitoring [14], and environmental monitoring [15]. As the main parameter for the quantitative study of surface urban heat islands (SUHIs), LST is an important data source for monitoring urban thermal environments [4]. At present, domestic and foreign scholars have made many achievements in the quantitative assessment of SUHI [16,17], the distribution characteristics of SUHI [18,19], and the analysis of SUHI influencing factors [20,21], based on LST. Studies have shown that the heterogeneity of urban and suburban surfaces, caused by the substitution of natural surfaces by large impervious surfaces during urbanization, is the fundamental reason for the formation of SUHI [22,23,24]. Urban land cover types [25,26,27,28] and urban forms [29,30] have direct driving effects on SUHI formation and development, while regional climate [31,32], human activities [29,33], and the built-up area [34] also affect the intensity and distribution of SUHI. However, these studies do not consider surface temperature directionality when calculating SUHI intensity by LST, thus measuring the urban thermal environment, which affects the accuracy of the studies to some extent. This problem of surface temperature directionality is called thermal radiation directionality (TRD). Because of the complex and diverse characteristics of urban surface structures, there are significant differences in the TRD. In highly urbanized areas, the daytime TRD can reach up to 10 K [35]. 

At present, research on TRD mainly adopts two methods: model simulations and observational tests. The observational tests are classified according to the observation height: ground-based, airborne, and satellite. In early tests on the surface temperature of the vegetation canopy, scholars found that the temperature varied with the viewing angle [36,37,38]. Moreover, an in-depth observational study on the vegetation TRD revealed that the vegetation TRD was significantly correlated with season, solar zenith angle, and solar azimuth [39], while it was also influenced by the component temperature, component emissivity, component proportion, and inter-component scattering effect [40]. As research on TRD progressed, observational tests on the urban surface were also carried out. However, because of the complexity of urban surface 3D structures, some observational tests have been conducted with thermal infrared imagers, in small study areas [41]. Ground-based observation platforms are more suitable for the TRD study of vegetation or small-scale urban surfaces, because of their narrow field-of-view coverage, and it is difficult to observe study areas with a large-scale and complex surface structure. With the development of airborne remote sensing technology, airborne platform tests compensate for the shortage of ground-based platforms in the coverage range of the field-of-view, and focus on the comparative study of the ground surface in different areas of the city. An airborne observation test by Lagouarde et al. [42] showed that the density of buildings has an important influence on the discrepancy in the directionality of surface thermal radiation in different areas of the city, with maximum TRD differences of up to 7 K. Jiang et al. [43] found that the maximum directional temperature difference in a residential area of Nanjing reached 14 K during the day and up to 3.5 K at night, in their tests observing the TRD. The TRD can reach up to 14 K in the center of Toulouse, France, during the winter [44], while the maximum temperature difference caused by the angle of observation exceeds 9.0 K in the center of Vancouver, Canada, during the summer [4]. Although airborne platforms solve the issue of a narrow field-of-view of ground observations, airborne tests are limited by observation time, space, and expense when studying a larger range of observation objects. Compared with ground and airborne observation platforms, satellites have the advantages of strong periodicity, high resolution, and wide global coverage; however, satellite sensor platforms that can simultaneously perform multi-angle observations are yet to be developed. Therefore, the directionality of satellite observations of LST is generally studied by satellite sensors with a wide field-of-view, such as ATSR, MODIS, AVHRR, and VIIRS. Among them, MODIS can conduct daily period observations of the Earth’s surface within the zenith angle observation range of −65° and 65°. However, MODIS does not acquire multi-angle LST instantaneously, and raw LST data cannot be used directly for TRD quantification. Hu et al. [45] proposed a method to obtain MODIS multi-angle LST data, which reduces the errors resulting from instantaneous field-of-view problems, by analyzing MODIS LST data in a long time series. On this basis, the effects of atmospheric attenuation and daily weather variability were eliminated by the difference in surface temperature and air temperature between urban land and adjacent urban water, and the LST related only to the observation angle was obtained. This study is the first of its kind to directly evaluate the effect of TRD on satellite-based SUHI measurements based on directional temperature and land-use intensity. The results show that TRD is up to 9 K during the day and is weaker at night, in addition to leading to an error of 2.3 K in SUHI intensity. Wang et al. [46] adapted Hu’s method, to evaluate the effect of weather variability and solar angle variability by model simulations, which simplified the method of estimating the TRD based on MODIS, but the model still needs further precision validation. 

Because of the heterogeneity and geometric structural characteristics of the urban surface, there are discrepancies in the thermal radiation received by different observation directions, resulting in a more significant TRD for the same observation. The TRD intensity also differs significantly under different land cover types; therefore, it is necessary to consider the TRD characteristics of each land cover type when conducting quantitative studies on SUHI intensity. However, the current urban heat island index calculation, using surface temperature without considering directionality, cannot truly reflect the actual situation of SUHI intensity. Moreover, there are various factors affecting the urban TRD, including observation time [44,47], surface geometric structure [48,49,50,51,52], physical properties of materials [49,53,54], and spatial locations between the sun-surface-sensor [55,56,57]; thus, most TRD studies selected more delineated partial surfaces of one or multiple cities for observation or model simulation, and rarely integrated the TRD with SUHI intensity.

Therefore, in this study, we quantified the TRD for different land-use intensities in Hefei for long time series (11 warm seasons) data by using the characteristics of the water body, approximating the Lambertian body to reduce the angular effect of atmospheric attenuation and the effect of daily temperature variations in LST. We also evaluated the influence of the TRD on the quantitative study of SUHI intensity, through a comparative analysis of the TRD for different land-use intensities. The significance of this study is to quantify the urban TRD in Hefei under different land-use intensities over a long time series, and to evaluate the impact of TRD on the results of SUHI intensity, based on satellite data, which provides a methodological case for quantifying urban TRD, to improve the accuracy of quantitative research on SUHI intensity.

## 2. Materials and Methods

### 2.1. Study Area

**Figure 1:**
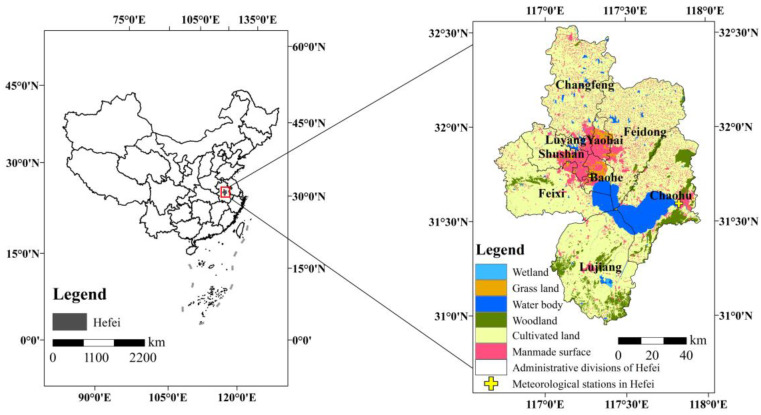
1 km pixel scale land-use types for Hefei (approximately 127 km × 178 km) in 2020.

Hefei, Anhui Province, China, was selected as the study area (Figure 1). Hefei, the capital of Anhui Province, is located between the Yangtze River and Huaihe River in the central part of Anhui Province. Its geographical range spans 116°41′–117°58′ E and 30°57′–32°32′ N [58], and it has a subtropical monsoon humid climate, with a clear monsoon season, four distinct seasons, mild climate, and overall moderate rainfall [59]. Chaohu Lake is located in central Anhui Province, surrounded by Hefei, Chaohu, Feidong, Feixi, and Lujiang, with a length of 55 km from east to west, a width of 21 km from north to south, a perimeter of 176 km, an average depth of 2.89 m, an area of 780 km^2^, and a volume of 2.07 billion m^3^; it is known as one of the five famous freshwater lakes in China [60,61]. Due to the continuous expansion of urban construction and the rapid growth of the total urban population in Hefei, the resource utilization and ecological environment in the area have been seriously affected, leading to significant changes in the nature of the underlying urban surface and a dramatic increase in anthropogenic heat emissions. While the rapid economic development of Hefei has led to increasingly prominent thermal environmental problems in Hefei. To evaluate the influence of TRD on SUHI intensity, this study analyzes the TRD variation characteristics of land-use intensity at all levels, under different satellite overpass times. Therefore, this study evaluated the entire jurisdiction of Hefei in the study (there are obvious differences in the level of urbanization among Shushan District, Baohe District, Luyang District, Yaohai District, Feixi County, Feidong County, Lujiang County, Changfeng County, and Chaohu City).

### 2.2. Data

MODIS sensors carry out periodic observations of the Earth’s surface using NASA’s Terra and Aqua polar-orbit satellites. The data acquired by MODIS sensors have high time-space-spectral comprehensive resolution, wide coverage, and free access; however, compared with airborne platform observation or quasi-simultaneous directional model simulation, it is difficult to perform multi-angle observations of the same ground object in an instantaneous field-of-view [62]. Therefore, this study selected long-term series statistics of LST data in Hefei, to reduce the observation error caused by the non-instantaneous field-of-view. MODIS daily LST products (MOD11_A1 and MYD11_A1) are the main data used for LST directionality assessment, and the four overpass times of the satellite (which can be queried by https://oceandata.sci.gsfc.nasa.gov/overpass_pred/ (accessed on 15 August 2022)) were 10:30 (Terra-day), 13:30 (Aqua-day), 22:30 (Terra-night), and 01:30 (Aqua-night) Beijing time. Moreover, the sensor zenith angle (SZA) data utilized in this study were derived from viewing angle data from MODIS daily LST products. According to the above requirements for experimental data, this study collected MODIS daily LST products for May–September 2010–2020. To reduce the effect of cloud occlusion pixels, such as cloud edges, the lowest LST threshold was set to 270 K and low-temperature anomalies were removed. Considering that the summer LST changes and directional differences are more obvious [47], to avoid the influence of seasonal changes in surface vegetation and differences in snow cover on the reflectance of various objects on the urban surface received by the sensor, this study selected May–September, over 11 years, as the observation period. In addition, this study is based on MODIS LST data, because the MODIS data are influenced by atmospheric attenuation factors, and considering the problem of daily temperature variation, the hourly air temperature data of Hefei land and Chaohu Lake, combined with MODIS LST data, were used to reduce the interference of atmospheric factors on urban TRD quantification (the air temperature data of Hefei land were taken from www.ncei.noaa.gov/maps/hourly/ (accessed on 29 December 2021) and the air temperature data of Chaohu Lake were obtained from the Anhui Meteorological Service). Finally, to calculate the influence of TRD on the quantitative study of SUHI intensity, this study selected the 2015 impervious surface cover data of Hefei as the base data of land-use intensity, and resampled the impervious surface cover data to the image element scale of 1 km, for coupling with MODIS LST data.

### 2.3. Methodology

The real surface is a non-Lambert body, and the urban surface has a complex three-dimensional structure; therefore, the surface component temperature and the difference in emissivity between components must be considered in the quantitative study of urban surface heat island intensity. A water body is similar to a Lambert body and shows a relatively uniform temperature distribution when observed from different angles [63,64]. The physical characteristics of a water body can be used to separate the components of MODIS LST products, other than directional factors such as the angle effect of atmospheric attenuation and daily weather variations.

#### 2.3.1. Separating the Angular Effect of Atmospheric Attenuation

**Figure 2:**
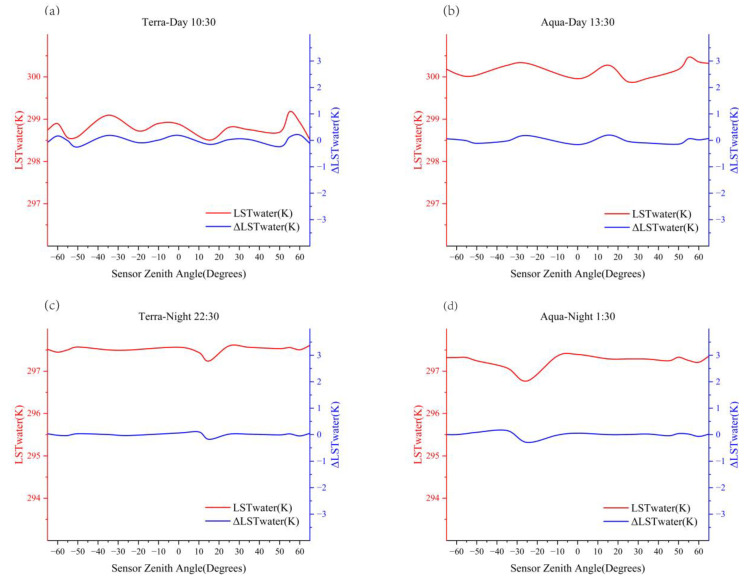
Distribution of $LST_{water}\left(t\right)$ (left *y*-axis, red) and $\;\Delta LST_{water}\;\left(t\right)$ (right *y*-axis, blue) at different sensor zenith angles (SZAs) (*x* axis), during each satellite overpass. The subfigures (**a**–**d**) respectively display the distribution of $LST_{water}\left(t\right)$ and $\;\Delta LST_{water}\;\left(t\right)$ at different SZAs during satellite overpass at 10:30, 13:30, 22:30, and 01:30.

The MODIS LST product used in this study was retrieved using the generalized split-window algorithm [65]. Compared with other retrieval algorithms, the algorithm eliminates the atmospheric effect, by the absorption difference of adjacent thermal infrared bands, when performing atmospheric correction, rather than directly using radiative transfer model parameters and atmospheric profile parameters; it is difficult to determine the source of atmospheric attenuation factors for LST [45]. Thus, MODIS LST data may be influenced by atmospheric attenuation, except for the TRD. In this study, to quantify the directionality of the TRD, the influence of atmospheric attenuation was mainly manifested in the interference of the angular effect of atmospheric attenuation. For the interference of atmospheric attenuation factors, we selected Hefei and Chaohu Lake, which are closely connected spatially, as the land surface and the water surface in this study. First, it can be assumed that the atmospheric conditions of both are similar, and the LSTs of land and water have similar atmospheric attenuation effects, under the same sensor zenith and azimuth angles. Second, based on this assumption, it is feasible to separate the interference of atmospheric attenuation through the difference between the LSTs of land and water, as well as the characteristics of the water body, without obvious TRD, as shown in Equation (1).

(1) $$\Delta LST\left(t\right)=LST\left(t\right)-\bar{\;\;LST_{water}\left(t\right)\;\;\;\;}$$

where t is the satellite overpass time, LST(t) is the LST of the satellite overpass time. (LST(t) is obtained by calculating the average value of all pixels over the land in Hefei, on a daily scale, during each overpass time over the study period. In addition, the LST(t) at different angles is obtained by calculating the average value of all land pixels in Hefei at that angle, on a daily scale, during each overpass time over the study period.) $\bar{LST_{water}\left(t\right)}$ is the average LST of all the water body pixels in the region, and ΔLST(t) is the difference between land LST and water LST. The complex three-dimensional structure of the land surface leads to differences in LST observed in different directions. Several steps are required to verify that the surface structure of the water body has no obvious directional characteristics, and to evaluate the feasibility of the method of eliminating the atmospheric attenuation angle effect using Equation (1). First, the $\Delta LST_{water}\left(t\right)$ is obtained, by counting the $LST_{water}\left(t\right)$ of different SZAs during each satellite overpass and combining with Equation (1). Second, the fluctuations in $LST_{water}\left(t\right)$ and $\Delta LST_{water}\left(t\right)$ at different angles are used to verify the TRD characteristics of the water body, and to evaluate the effectiveness of this study in separating atmospheric attenuation interference. In this study, we found that the MODIS sensor covered a maximum viewing angle of 12° when overpassing Hefei and cannot cover the entire study area range; thus, it is important to note the difference in the SZAs when analyzing changes in ΔLST(t) at different angles. To solve this problem, this study divides all SZAs into one group every 5°, and $LST_{water}\left(t\right)$ in each group is averaged as $\bar{LST_{water}\left(t\right)}$, to analyze the influence of SZA difference on $\Delta LST_{water}\left(t\right)$ (because for some overpass times the viewing angles of Chaohu water are incomplete, some intervals are averaged at intervals of 10°). As shown in Figure 2, the changes in $LST_{water}\left(t\right)$ (red line, left *y*-axis) and $\Delta LST_{water}\;\left(t\right)$ (blue line, right *y*-axis) in the Chaohu Lake waters of Hefei, during each satellite overpass, show small fluctuations and are generally stable. This shows that the LST of Chaohu Lake has a relatively uniform distribution, with a maximum difference of 0.46 K when observed at different SZAs, which is consistent with the observed results of TRD on the sea surface [47]. Therefore, the more homogeneous surface structure of the water body has no obvious TRD characteristics, and it is feasible to use the uniform surface structure characteristics of the water body to eliminate the atmospheric attenuation angle effect factors. However, the fluctuations in $LST_{water}\left(t\right)$ and $\Delta LST_{water}\;\left(t\right)$ are highly correlated in Figure 2, and the fluctuation in $\Delta LST_{water}\;\left(t\right)$ is smaller than that in $LST_{water}\left(t\right)$. The influence of atmospheric attenuation angle effect of LST pixels in MODIS water can be effectively reduced by Equation (1), for the same sensor zenith and azimuth angle. As mentioned above, land and water pixels have the same atmospheric attenuation effect under the same or similar sensor angles. Therefore, this method can be applied to land pixels. Theoretically, the distribution of $\Delta LST_{water}\;\left(t\right)$ will be almost uniform after eliminating the atmospheric attenuation angle effect, but the distribution of $\Delta LST_{water}\;\left(t\right)$ still has small fluctuations, as shown in Figure 2. This shows that the influence of atmospheric factors cannot be completely separated by Equation (1) alone.

#### 2.3.2. Separating the Effect of Daily Weather Variations

In this study, the statistics of $\Delta LST_{water}\;\left(t\right)$ under different viewing sensor zenith angles, show that the sensor cannot cover all viewing zenith angles during the satellite overpass. Moreover, we found that the coverage of SZAs varies during the daily satellite overpass, and a larger zenith angle range corresponds to more observational data (the range of the SZA coverage also affects the number of clear-sky data for this zenith angle group). Therefore, the zenith angles of different coverage ranges will have different clear-sky data values, which will directly affect the LST under the zenith angles. In addition, according to the physical properties of land and water, the diurnal variation in water temperature is smaller than that of land; thus, daily weather variability has a greater impact on ΔLST in land areas. To reduce the effect of daily weather variability, and to solve the problem of differences in the distribution of clear and cloudy days between the SZA groups in the directional quantization process, this study introduced near-surface meteorological data from land and adjacent water (meteorological station locations are shown in Figure 1), to correct the effect of daily weather variations, as shown in Equation (2).

(2) $$\Delta T_{air}\left(t\right)=T_{air,land}\left(t\right)-T_{air,water}\left(t\right)$$

where $\Delta T_{air}\left(t\right)$ is the air temperature difference between the land of Hefei and Chaohu water during each satellite overpass, $T_{air,land}\left(t\right)$ is the air temperature of land during each satellite overpass ($T_{air,land}\left(t\right)$ is obtained by calculating the daily average air temperature of the land, during each overpass time, in each year over the study period), and $T_{air,water}\left(t\right)$ is the air temperature of water during each satellite overpass ($T_{air,water}\left(t\right)$ is obtained by calculating the daily average air temperature of the water, during each overpass time, in each year over the study period). The air temperature data in this study were derived from the records of regional near-surface meteorological stations, which were not affected by TRD, and there was a strong correlation between the air temperature and surface temperature data [66,67]. Thus, this study is feasible for solving the impact of surface temperature variations by analyzing air temperature variations. In addition, considering the radiation temperature, the physical mechanism of air temperature, and differences in land and water observation locations, daily weather variations may represent only a portion of the daily variations in ΔLST in Hefei. Thus, this study introduces the influence coefficient α, and uses Equation (3) to separate the impact of this factor.

(3) $$\Delta LST_{\Delta T_{air}}\left(t\right)=\Delta LST\left(t\right)-\alpha \times \left(\Delta T_{air}\left(t\right)-\bar{\Delta T_{air}}\right)$$

where ΔLST(t) is the difference between land and water LST during each satellite overpass (ΔLST(t) is calculated on a daily basis as the difference between the average LST of all land pixels and all water pixels in Hefei, during each overpass time, in each year over the study period), $\Delta LST_{\Delta T_{air}}\left(t\right)$ is ΔLST(t) after reducing the influence of daily weather variations, $\Delta T_{air}\left(t\right)$ is the difference in air temperature between land and water during each time of satellite overpass, $\bar{\Delta T_{air}}$ is the average value of $\Delta T_{air}\left(t\right)$ at four different times of satellite overpass, and α is the influence coefficient of daily weather variations on the daily variations in ΔLST.

**Figure 3:**
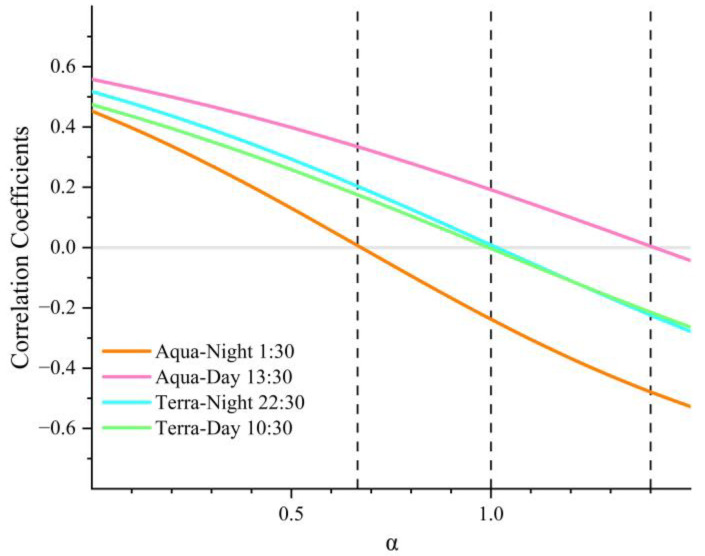
Pearson product moment correlation coefficient between $\Delta LST_{\Delta T_{air}}$ and $\Delta T_{air}$ at different α values. The abscissa is the value of α and the ordinate is the correlation coefficient between $\Delta LST_{\Delta T_{air}}$ and $\Delta T_{air}$.

In Figure 3, we find that $\Delta LST_{\Delta T_{air}}$ and $\Delta T_{air}$ show a correlation of approximately 0.5 for different satellite overpass times when α is 0 in Equation (3), during the 11 year study period, which verifies that there is a correlation between LST and air temperature. Therefore, it is feasible to reduce the effects of daily weather variations using Equation (3). In this study, the value of α is used as an adjustment factor, to account for the effect of daily air temperature variability on LST. The α values were determined by evaluating the correlation between $\Delta LST_{\Delta T_{air}}$ and $\Delta T_{air}$ at different values. The final α value was determined by identifying when the correlation between $\Delta LST_{\Delta T_{air}}$ and $\Delta T_{air}$ was minimized in the time series. Specifically, α values of 1, 1.4, 1, and 0.7, were found to minimize the correlation between $\Delta T_{air}$ and $\Delta T_{air}$ for Terra-day, Aqua-day, Terra-night, and Aqua-night, respectively, thus minimizing the effect of daily air temperature variability. In addition to the intuitive effect of daily temperature variations, other factors, such as surface humidity and wind, also affect the $\Delta LST_{\Delta T_{air}}$. However, because the analysis of $\Delta LST_{\Delta T_{air}}$ was highly aggregated in time and space in the later stage of this study, it reduced the influence of variations in other factors on our results.

#### 2.3.3. Relationship between SUHI Intensity and TRD

In previous sections, a method to reduce the interference of atmospheric factors was discussed. Furthermore, the $\Delta LST_{\Delta T_{air}}$ was obtained by reducing the atmospheric attenuation angle effect, daily weather variations, and other factors. To evaluate the influence of the TRD on the SUHI intensity in Hefei, this section introduces the maximum temperature difference between different SZAs, $\delta LST_{dir,max-min}$ (see Equation (4)), as the effective thermal anisotropy [49] (also referred to as TRD in this study). Additionally, the urban heat island curve (UHIC) method [68] is introduced, based on the effective thermal anisotropy. The UHIC method can be used not only to explore the spatial relationship between LST and urban land-use intensity, but also to describe the temperature variations in the spatial aggregation of different gridded land-use data. First, we used the impervious surface percentage grid data, at the 1 km pixel scale, of Hefei as the basis for the division of SUHI intensity, and divided all grid units in Hefei into nine categories, except Chaohu Lake. The pixels under each category had corresponding LST values. Finally, we divided all the SZAs into seven groups to calculate each group’s maximum SUHI value, to evaluate the impact of the TRD on the SUHI in Hefei.

(4) $$\delta LST_{dir,max-min}=max\left(\Delta LST_{\Delta T_{air}}\left(\theta \right)\right)-min\left(\Delta LST_{\Delta T_{air}}\left(\theta \right)\right)\;\;\;$$

where $\delta LST_{dir,max-min}$ is the maximum temperature difference at different SZAs. As the sensor azimuthal variations in this study were small, only the variations in zenith angles should be considered for $\delta LST_{dir,max-min}$. $max\left(\Delta LST_{\Delta T_{air}}\left(\theta \right)\right)$ and $min\left(\Delta LST_{\Delta T_{air}}\left(\theta \right)\right)$ represent the maximum and minimum values of $\Delta LST_{\Delta T_{air}}$ observed at different SZAs, respectively.

## 3. Results

### 3.1. Spatial and Temporal Distribution Characteristics of TRD

**Figure 4:**
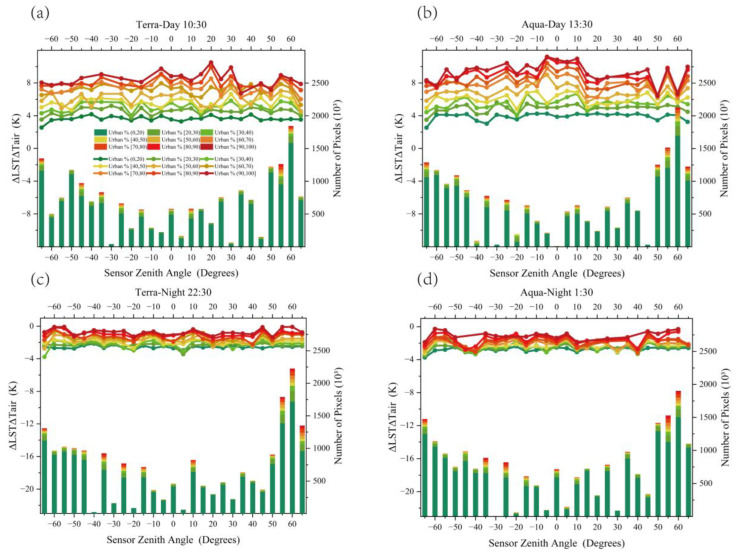
Statistical distribution of average $\Delta LST_{\Delta T_{air}}$ of each zenith angle, under different land-use intensities, at each satellite overpass time for Hefei. The right *y*-axis is the number of pixels with different land-use intensities of each SZA (represented by a histogram); the left *y*-axis is the average $\Delta LST_{\Delta T_{air}}$ of each SZA, under different land-use intensities (represented by a line chart). The subfigures (**a**–**d**) illustrate the statistical distribution of the average $\Delta LST_{\Delta T_{air}}$ for each zenith angle under different land-use intensities during satellite overpass at 10:30, 13:30, 22:30, and 01:30, respectively.

In this study, the land-use intensity was divided by the impervious surface percentage grid data of Hefei, at the 1 km pixel scale, and the $\Delta LST_{\Delta T_{air}}$ of different SZAs at each overpass time was calculated, and the results are shown in Figure 4. To ensure the statistical significance of the data and avoid the contingency and outliers caused by the small number of pixels, this study set the pixel number threshold to 100 when counting the pixel number of each SZA for different land-use intensities.

It can be seen from Figure 4 that $\Delta LST_{\Delta T_{air}}$ changes more strongly during the day than at night, and an obvious $\Delta LST_{\Delta T_{air}}$ hotspot appears at 10:30, when the SZA is 20° (see Figure 4a), and this hotspot is more obvious in areas with higher urban land-use intensity. Moreover, we found that the SZA was approximately the same as that of the sun at this time, but the position of the sun and the sensor were not on the same side. The appearance of this hotspot was most likely caused by the higher proportion of the sunlight surface observed under the 20° zenith angle compared to other zenith angles. In addition, another hotspot appeared at 13:30, when the sensor was near the zenith position (see Figure 4b). The reason for this, is that the sensor and sun were located on the same side at this time, and the sunlight surface accounted for a relatively high proportion of the response. Notably, the SZA was near the nadir at this time; compared with other SZAs, the roof and street components in the field-of-view accounted for a relatively high proportion of the response. Meanwhile, the roof and part of the street were affected by sunlight for a long time, combined with the heat accumulation of building materials, this resulted in localized high temperatures on the ground surface under this SZA. Studies on the roof, street, and wall component temperature comparisons can be found in [49,53]. In addition, the $\Delta LST_{\Delta T_{air}}$ fluctuation was more obvious when the daytime SZA was close to the maximum, and the proportion of the wall component in the field-of-view was higher at this angle. Compared with roofs, walls were vulnerable to the shadow of other buildings and the influence of the solar position, and the distribution of sunlight was more complex, while wall heat increase was not as obvious as that of roofs, streets, and other components. Therefore, $\Delta LST_{\Delta T_{air}}$ under a larger SZA during the day was relatively low, and there was also a large difference between the wall-to-wall temperatures for different times and proportions of sunlight surface, which is essentially the same as the results of the multi-angle observations in Toulouse [42]. At night, the $\Delta LST_{\Delta T_{air}}$ distribution of each SZA was significantly different from that of the daytime, and the overall distribution characteristics were low in the middle, and high on both sides (Figure 4c,d). This distribution characteristic was mainly caused by the high proportion of wall components in the field-of-view, when the SZA was large at night, and the thermal insulation effect of walls outperformed those of roofs and streets [69,70]; thus, the overall nighttime temperature was higher at this SZA. 

**Figure 5:**
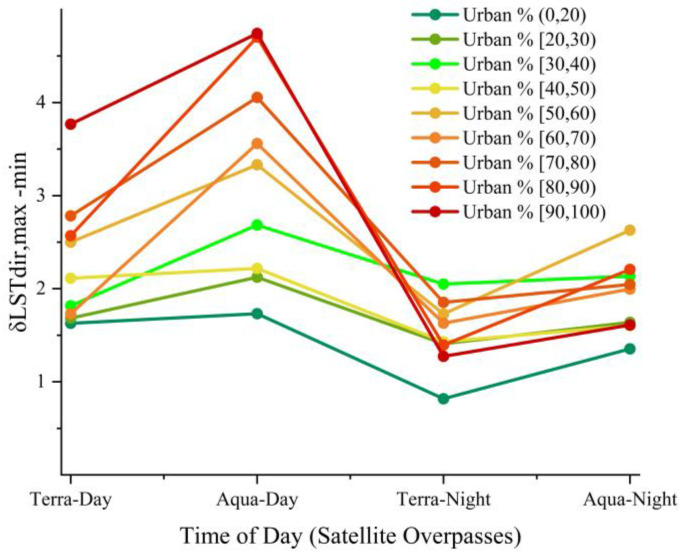
Effective thermal anisotropy under different land-use intensity groups, at each satellite overpass time. The abscissa represents the four overpass times, the ordinate represents the effective thermal anisotropy, and the colored solid line represents the effective thermal anisotropy of the land-use intensity groups.

As shown in Figure 5, the maximum TRD of Hefei reaches 4.7 K, which appears in the land-use intensity group with the highest degree of urbanization, in the afternoon. This result is consistent with the research results of TRD in Chicago and New York [45], both spatially and temporally; however, there are significant differences in TRD intensity. This is closely associated with the level of urbanization development in the study area, the shape of the surface structure of the study area, and differences in the climatic environment. To further understand the differences in urban surface structure among Chicago, New York, and Hefei, this study compared and analyzed Google Earth and building height data for these cities. The results show that the low-rise buildings in Chicago and New York are densely and mostly regularly distributed, in residential areas and other land types with relatively low economic value, whereas the high-rise buildings show relatively scattered and irregular distribution, mostly concentrated in urban economic centers. Within the time range of this study, the urban surface of Hefei was mostly composed of densely packed middle- and high-rise buildings, with block distribution, and relatively low-rise buildings with a dense and regular distribution. Most middle- and high-rise buildings in Hefei are widely distributed, and are found in residential areas built around commercial areas, which is caused by the large population and shortage of land in Hefei, resulting in high-rise residential areas. Combined with the above differences in TRD intensity, there is a significant connection between the height of urban buildings and the density of their distribution and TRD. Regarding the influence of the three-dimensional structure of urban surfaces on TRD, scholars have obtained similar results through models or observations, in which urban landscapes with medium-density high-rise buildings, show higher TRD [49,52,71]. 

### 3.2. Influence of TRD on SUHI Quantification

**Figure 6:**
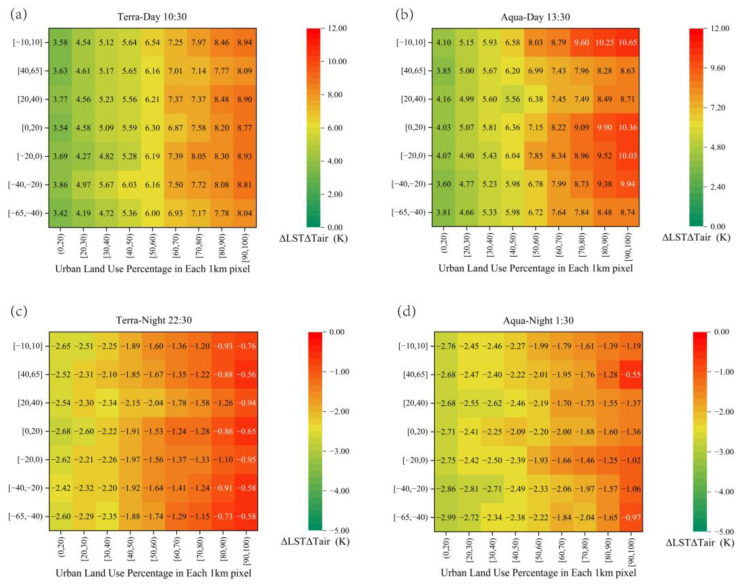
Heat map of $\Delta LST_{\Delta T_{air}}$ distribution for each SZA group at different land-use intensity percentage, for the four overpass times. The abscissa represents the land-use intensity percentage in a 1 km grid cell, and the ordinate represents the SZA group. Specifically, the subfigures (**a**–**d**) illustrate the distributions of $\Delta LST_{\Delta T_{air}}$ for each SZA group at different land-use intensity percentages during satellite overpass at 10:30, 13:30, 22:30, and 01:30, respectively.

In this study, all SZAs were divided into seven groups of SZA ranges, and the $\Delta LST_{\Delta T_{air}}$ for each SZA group was counted, based on $\Delta LST_{\Delta T_{air}}$ under different land-use intensity percentages, as shown in Figure 6. The $\Delta LST_{\Delta T_{air}}$ in the afternoon, near the zenith position, was larger than the angle groups at other times and positions. This distribution feature was mainly caused by the different sunlight and shadow conditions of the observed objects at different sensor-sun-ground positions, as well as the different component area proportions of the surface, roof, wall, etc., in the field-of-view of the SZA group. At nighttime, we found that the distribution of $\Delta LST_{\Delta T_{air}}$ was the opposite of that in the daytime, with the larger viewing angle group (when the SZA in this group was close to maximum) being higher than in the other groups. This distribution feature occurs because, when the conditions of sunlight and shadow components are relatively uniform, the main source of temperature variation is related to the thermal inertia of the materials of urban surface attachment and the anthropogenic thermal contribution inside the buildings. For example, in the [−65, −40] zenith angle group in Figure 6c,d, when the sensor observation location is close to the ground, the component of the wall within the sensor has a higher areal proportion compared to the other components, and the wall is warmer than the roof and street because of the difference in the insulation properties of the building material [35,69,70]. Therefore, $\Delta LST_{\Delta T_{air}}$ fluctuated more smoothly at night. Moreover, the temperature variation characteristics of the zenith angle group were opposite to those in the daytime.

**Figure 7:**
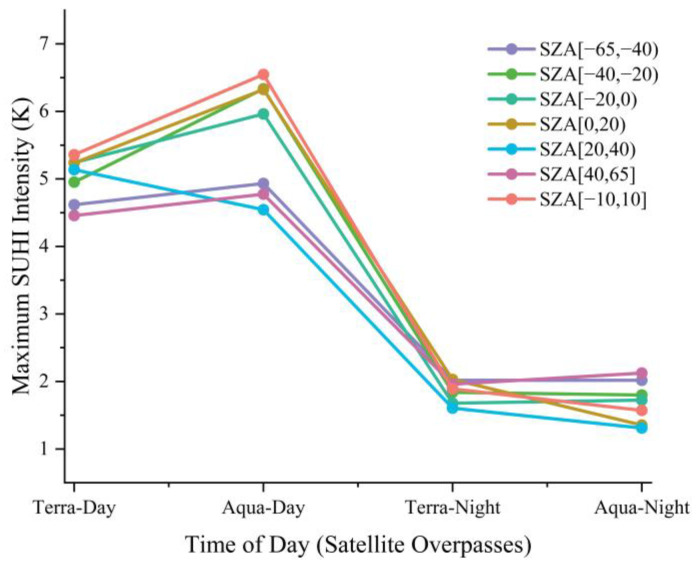
Line graph showing the maximum SUHI of each SZA group during each sensor overpass, the horizontal axis represents the four overpass times, the vertical axis represents the maximum SUHI, where the colored solid line is the variations in the maximum SUHI of each SZA group with respect to the satellite overpass time.

Based on the statistics of $\Delta LST_{\Delta T_{air}}$ for each zenith angle group, this study directly evaluated the influence of TRD on SUHI quantification, and the results are shown in Figure 7. The SUHI was higher in areas with high land-use intensity percentages and developed urbanization levels, than in other areas. In addition, in Figure 7, there is a more significant difference in temperature for the same land-use intensity percentage under different SZA groups, which is mainly caused by the influence of TRD, and generates up to 2.0 K uncertainty for quantifying the SUHI in Hefei, which is approximately 31–44% of the total SUHI. Moreover, the impact of TRD during the daytime was more significant than that at nighttime, averaging approximately 1.37 K, compared to only 0.61 K at night. This is roughly the same as in the study of urban TRDs in New York and Chicago [45]. Despite a certain demonstration of the generalizability of using water bodies to separate disturbances and thus quantify TRD, the results are still not directly applicable to the quantification of global heat island intensity, as the key factor is the significant differences in the surface structure and geo-climatic environments of different cities.

## 4. Discussion

This study conducted a comprehensive analysis of the TRD in Hefei, based on eleven years of MODIS LST data, and reduced the angular effect of atmospheric attenuation and the effect of daily temperature variations in quantifying the TRD, and finally evaluated the impact of TRD on the quantification of SUHI intensity, observed by satellite. However, the present study has room for further improvement. In this study, a land-use intensity division was constructed based on impervious surface data, and the variability characteristics of TRD under different land-use intensities were analyzed. However, because of the difficulty in obtaining 3D morphological data (such as the ratio of urban building height and street width) in Hefei, a specific analysis of the relationship between the urban 3D morphological ratio and the spatial and temporal variation patterns of urban TRD and hotspot distribution, needs to be further developed. Land-use types have a significant impact on the distribution of TRD. Different types of land use, such as buildings, roads, green spaces, and farmland, exhibit distinct abilities to reflect, absorb, and conduct solar radiation, thereby impacting the directional distribution of LST. In this study, the prevalence of farmland in the study area, relative to cities such as New York and Chicago, with less farmland, leads to a lower overall SUHI and TRD in Hefei. To enhance our comprehension of the changes in TRD resulting from varying land-use intensities, a quantitative investigation of the impact of specific land-use types on TRD should be conducted in the future. This can be achieved by subdividing land-use types and identifying the differences and patterns in TRD for each type. Moreover, by comparing the TRD intensity with the SUHI of different land-use types, we can obtain a more precise evaluation of the influence of land-use types on the formation of urban heat islands. To address the problem of MODIS’s non-instantaneous field-of-view observations, this study analyzes long time series of MODIS LST data, to decrease the uncertainty caused by the instantaneous field-of-view problem and to ensure accuracy in quantifying the TRD. In the future, this problem can be addressed by methodological improvements. For example, the sensor field-of-view is restricted to a narrow range, in which all pixels are observed at the same overpass time [72,73]. Finally, for the analysis of the results, we find that in the comparative analysis of the urban TRD in Hefei, New York, and Chicago, there are significant differences in the intensity of directionality among the three cities, but there are similarities in the distribution of directional hotspots, and the characteristics of variation in directionality under different land-use intensities. These similarities may primarily be attributed to the similarities in the layout of urban structures, population density, and building materials among the three cities. Additionally, the similar climatic conditions among the three cities may contribute to this phenomenon. To investigate whether different urban study areas have the commonality of directional hotspot distribution and variation characteristics, we will conduct a comprehensive study and analysis of multiple cities in different geographical locations globally, in the future, and thus explore the pattern of TRD hotspot distribution.

## 5. Conclusions

Based on the problem that TRD interferes with the quantification of SUHI intensity, this study collates and counts MODIS LST data for the warm months (May–September) in Hefei, over eleven years. Based on the differences in surface materials between land and adjacent water bodies, the similarity of daily weather variations, and the similarity of the angular effect of atmospheric attenuation, we separated atmospheric attenuation and daily weather variations from the MODIS LST data, to quantify the TRD at each land-use intensity. Finally, we evaluated the influence of the TRD on the SUHI based on satellite data, by comparing the SUHI of each SZA group. The results show that: (1) there is a significant diurnal difference in the urban TRD, and the TRD in the highest urbanized areas can reach up to 4.6 K during the daytime, while it is relatively low at night, but still up to 2.6 K. This result is similar to the results of aerial observations and urban TRD model simulations, in terms of the spatial and temporal distribution characteristics of TRD. (2) During the daytime, two directional “hot spot” features are observed: one is in the forenoon, where the SZA is approximately the same as the solar zenith angle, but the sensor and sun are positioned on opposite sides. The other is in the afternoon, when the SZA is near the nadir, and the sensor and sun positions are on the same side. The causes of both hotspots are related to the proportion of the sunlight shadowing component. (3) Urban morphological characteristics, local land and water layouts, and climatic environment are important factors that cause differences in the intensity of TRD among cities. (4) The effect of the TRD on SUHI intensity, quantified based on satellite data in Hefei, is approximately 2.0 K, which is approximately 31–44% of the total SUHI in Hefei. This study quantitatively evaluates the influence of urban TRD on SUHI and provides theoretical guidance for quantitative and accurate remote sensing estimation of the urban heat island, which is expected to promote the development of quantitative urban environment thermal remote sensing monitoring.

## Data Availability

The data used to support the findings of this study area are available from the corresponding author upon request via email.
